# Genetically determined circulating micronutrients and the risk of nonalcoholic fatty liver disease

**DOI:** 10.1038/s41598-024-51609-3

**Published:** 2024-01-11

**Authors:** Ke Liu, Ying Chen, Jiaxin Chen, Weiwei Chen, Xiaohui Sun, Yingying Mao, Ding Ye

**Affiliations:** School of Public Health, Zhejiang Chinese Medical University, 548 Binwen Road, Hangzhou, 310053 Zhejiang China

**Keywords:** Metabolic disorders, Risk factors

## Abstract

Evidence from epidemiological literature on the association of circulating micronutrients with risk of nonalcoholic fatty liver disease (NAFLD) is inconsistent. We aimed to elucidate the causal relationships using Mendelian randomization (MR). Single-nucleotide polymorphisms associated with 14 circulating micronutrients (β-carotene, calcium, copper, folate, iron, magnesium, phosphorus, selenium, vitamin B6, B12, C, D, K1 and zinc) were employed as instrumental variables. Summary level data for NAFLD were obtained from a genome-wide association study (GWAS) meta-analysis of 8434 cases and 770,180 controls (discovery stage) and another two datasets including 1483 NAFLD cases and 17,781 controls (replication stage 1) and 2134 NAFLD cases and 33,433 controls (replication stage 2). Inverse variance-weighted method (IVW) was used as primary analysis, supplemented with a series of sensitivity analysis. Genetically predicted higher β‑carotene levels were suggestively associated with reduced NAFLD risk [odds ratio (OR) 0.81, 95% confidence interval (CI) 0.66–0.99; *P* = 0.047], whereas the association did not survive the false discovery rates (FDR) correction (*P*_FDR_ = 0.164). Genetically predicted circulating iron (OR 1.16, 95% CI 1.05–1.29; *P* = 0.006, *P*_FDR_ = 0.028), selenium (OR 1.11, 95% CI 1.03–1.20; *P* = 0.005, *P*_FDR_ = 0.028) and vitamin B12 (OR 1.08, 95% CI 1.03–1.13; *P* = 0.002, *P*_FDR_ = 0.028) were significantly associated with increased risk of NAFLD. Moreover, the findings were consistent in individual datasets (*P*_heterogeneity_ > 0.05) and confirmed in sensitivity analysis. Our study provided evidence that circulating iron, selenium and vitamin B12 might be causally linked to the risk of NAFLD, which deserves further exploration of the potential biological mechanism.

## Introduction

Nonalcoholic fatty liver disease (NAFLD) is a burgeoning public health concern, and has reached the pandemic level with a recent prevalence estimate of 29.8% worldwide^1^. Alarmingly, the prevalence and mortality of NAFLD is projected to continuously increase in Western countries as well as in several Asian areas by 2030^2^. Currently, the pathogenesis of NAFLD is framed in the ‘multiple-hits hypothesis’ where a number of diverse parallel processes may contribute to the development and progression of liver inflammation^3^. As the principal contributory factor of extrahepatic factors, nutrition has been reported to affect NAFLD pathogenesis in multiple publicaitons^4^. At present there is no clear consensus on the pharmacological treatment of NAFLD, therefore, nutrition management as an important modifiable lifestyle remains the first line of prevention strategy of NAFLD^5^.

A growing number of studies have shown that the development and severity of NAFLD is closely related to micronutrients such as circulating selenium, iron, vitamin B12, and vitamin D^6–8^. For example, vitamin D deficiency was found to be associated with the presence and severity of NAFLD in a meta-analysis of cross-sectional and case–control studies^9^. However, the causality and clinical significance of this association remain undetermined, since NAFLD may lead to a decrease in circulating 25(OH)D by decreasing 25-hydroxylase activity^9^. Moreover, the negative association may be attributed to confounding factors including obesity, insulin resistance and metabolic syndrome^10^. Thus, the casual role of micronutrients in the development of NAFLD remains unclear.

Mendelian randomization (MR) is a specific type of instrumental variable (IV) analysis that uses genetic variation as IV to detect and quantify potential causal relationships between exposure and disease. The approach carries two merits of minimizing confounding and diminishing reverse causality because genetic variants are randomly allocated at conception and cannot be modified by the development and progression of the disease^11,12^. Here, we use recent large summary statistics for NAFLD and circulating micronutrients to comprehensively investigate the relationship of circulating micronutrients with NAFLD using two-sample MR methods.

## Methods

### Overview

**Figure 1:**
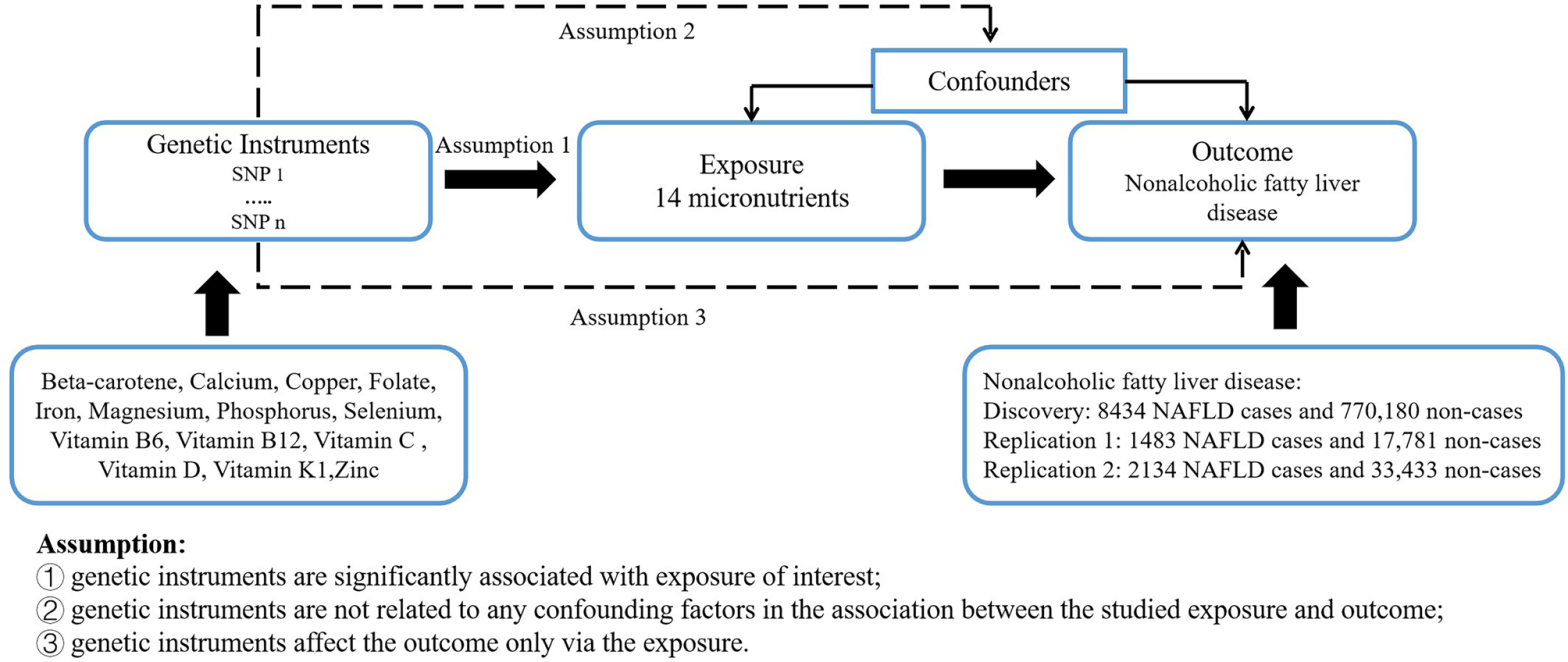
Overview of the Mendelian randomization (MR) design.

A schematic summary of the study design is given in Fig. 1. Firstly, we examined the associations of micronutrients with NAFLD in a large discovery dataset and then performed replication analysis in two independent datasets using two-sample MR approach. To increase statistical power, we combined estimates from these data sources. All studies included in the cited genome-wide association studies (GWASs) had been approved by relevant review boards. No additional ethics approval or consent to participate was required.

### Outcome data sources

Summary-level data for NAFLD were extracted from a GWAS meta-analysis (the Electronic Medical Records and Genomics, UK Biobank, FinnGen, and Estonian Biobank) including 8434 NAFLD cases and 770,180 controls (discovery stage)^13^ and another two GWASs including 1483 NAFLD cases and 17,781 controls (replication stage 1)^14^ and 2134 NAFLD cases and 33,433 controls (replication stage 2)^15^ (Table S1). Case definition criteria of included NAFLD GWASs are shown in Table S1. Detailed information on quality control refers to the cited GWAS papers.

### Selection of instrumental SNPs

We conducted a search of the latest published GWASs performed among European individuals on circulating micronutrients. A total of 14 nutrients were finally retrieved, namely β-carotene^16^, calcium^17^, copper^18^, folate^19^, iron^19^, magnesium^20^, phosphorus^21^, selenium^22^, vitamins B6^23^, B12^24^, C^25^, D^26^, K1^27^ and zinc^18^ (Table S1). Vitamins A^28^ and E^29^ were excluded because the corresponding GWASs were adjusted for body mass index (BMI), which may bias MR estimates. In terms of calcium, phosphorus and vitamin D, more recent GWASs that have been conducted in UK Biobank^30^ were not used, due to sample overlap with outcome data sources. We selected eligible genetic instruments based on the following criteria that single nucleotide polymorphisms (SNPs) should independently (r^2^ < 0.01) affect the concentrations of these nutrients at a genome wide significance level (*P* < 5 × 10^−8^). For nutrients instrumented by less than two SNPs, suggestive significant genome-wide association significant (*P* < 1 × 10^–5^) or validated SNPs were included if available. For serum iron, we used four instrumental SNPs (rs1800562, rs1799945, rs855791 and rs57659670) as their concordant association with the other three biomarkers (ferritin, transferrin saturation and total iron binding capacity) of systemic iron status. To further identify the rationality for our IV selection, the plausible biological interpretation of the 71 SNPs were identified using g:Profiler (https://biit.cs.ut.ee/gprofiler/snpense, accessed on 11 Dec 2023), and 68 snp-to-gene were identified. For example, the SNPs associated with iron, rs1799945 and rs1800562 are localized in the gene HFE, rs57659670 is localized in the gene DUOX2, and rs855791 is localized in the gene TMPRSS6. The HFE protein functions to regulate iron absorption by regulating the interaction of the transferrin receptor with transferrin^31^. DUOX2, as a member of the NADPH oxidase family, has been demonstrated to be involved in the production of ROS to promote ferroptosis^32^. TMPRSS6 is a transmembrane serine protease primarily expressed in hepatocytes, which is a critical negative regulator that controls hepcidin transcription^33^. Among the instrumental SNPs associated with selenium, four out of five SNPs were localized in the gene DMGDH. This gene encodes an enzyme involved in metabolism of sulfur-containing amino acids (conversion of homocysteine to methionine) and may therefore play a role in selenoamino acid metabolism^18^. The detailed information of selected genetic variants used in the present study are listed in Table S2.

### Statistical analysis

The random-effects inverse-variance weighted (IVW) method was used to evaluate the potential causal relationship of circulating micronutrients with risk of NAFLD as primary analysis. IVW method combines the causal effect estimates of multiple single IVs by Wald ratio method^34^. Cochran’s Q statistic was used to assess the heterogeneity of SNP-estimates in each MR association. Additionally, we performed the MR-Egger regression, maximum likelihood-based, simple median, weighted-median and MR-Steiger sensitivity analyses, which are more robust to the inclusion of pleiotropic instruments. The MR-Egger regression is disposed to regression dilution bias, and the average horizontal pleiotropic effect across all genetic variants can be interpreted by the intercept term^35^. Specifically, the intercept did not significantly differ from 0 (*P* > 0.05), pleiotropic effects were considered absent. Furthermore, the maximum-likelihood method was used to validate the result from the IVW method, which was assessed by assuming that there was a linear relationship between circulating micronutrients and risk of NAFLD^36^. Simple- and weighted-median methods was used estimate the potential cause effects when IVs went against standard assumptions. The weighted median method can obtain consistent estimation of the causal effect as long as the weight of the causal effect calculated by the effective IV reaches 50%^37^. In addition, we used the MR pleiotropy residual sum and outlier (MR-PRESSO) test to identify the possible pleiotropic outliers and reassessed the causal effect estimates after removing outliers^38^. Finally, the MR‐Steiger directionality test was used to determine whether the assumption that exposure causes outcome is valid^39^. The F statistic was calculated to measure the strength of used instruments and power was estimated using an online tool (https://cnsgenomics.shinyapps.io/mRnd/)^40^.

Random-effects meta-analysis was conducted to combine MR estimates from the three different data sources, along with the heterogeneity test. The Benjamini–Hochberg method was used to control the false discovery rate (FDR) for multiple testing, and adjusted *P* value ≤ 0.05 was regarded as statistically significant. *P* values below 0.05 but not survived the FDR correction were considered as suggestive association. All tests were performed using the Mendelian Randomization and TwoSampleMR packages in R software version 4.2.0 (R Foundation for Statistical Computing, Vienna, Austria).

## Results

**Figure 2:**
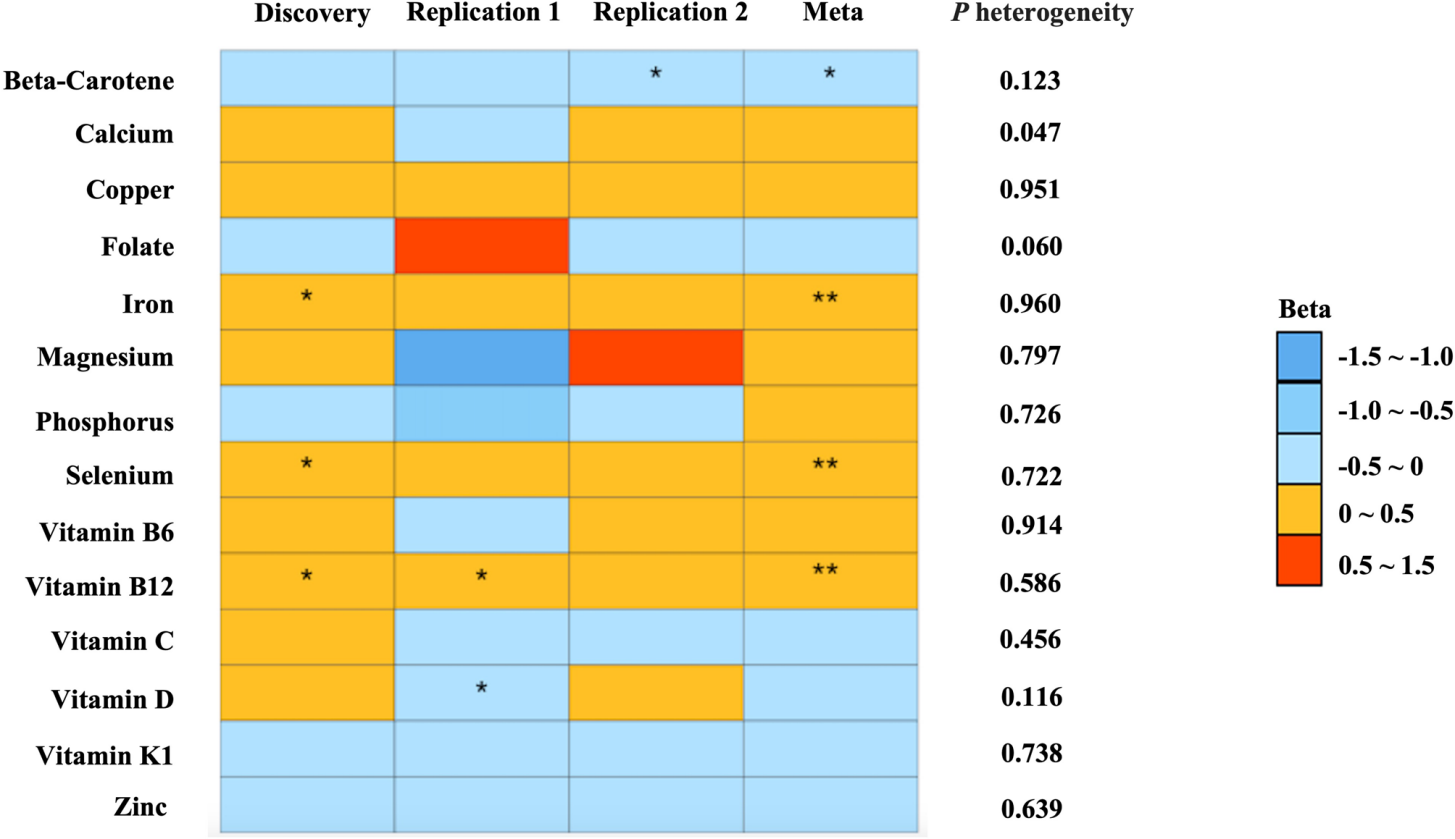
Heatmap of the associations of the 14 micronutrients with the risk of NAFLD from the inverse variance weighted (IVW) method. One asterisk (*) indicates the suggestive evidence for a potential causal association, while two asterisks (**) denote that the associations were statistically significant after the false discovery rates (FDR) correction in meta-analysis.

The F statistic for instruments and estimated power for all analyses are shown in Table S3. All F statistics for the IVs of each micronutrient were over 10, indicating a good strength of used genetic instruments. Overall, we found four micronutrients were associated with the risk of NAFLD in combined MR analyses. There was no evidence of heterogeneity in these associations (Fig. 2), and the directionality was confirmed by the MR-Steiger test (*P* < 0.001, Table S4). After FDR correction, genetically predicted iron, selenium and vitamin B12 with NAFLD reached statistical significance (Table 1).

**Table 1:** Associations of circulating micronutrients with risk of NAFLD by meta-analysis of discovery and replication datasets.

Nutrients	Inverse-variance weighted	P value^a^	P FDR value	MR-Egger	Weighted median	Simple median	Maximum-likelihood method	MR-PRESSO
	OR (95% CI)			OR (95% CI)	OR (95% CI)	OR (95% CI)	OR (95% CI)	OR (95% CI)
Beta-Carotene	0.81 (0.66, 0.99)	0.047	0.164	0.88 (0.54, 1.43)	0.82 (0.68, 0.98)	0.71 (0.47, 1.07)	0.81 (0.67, 0.99)	–
Calcium	1.17 (0.83, 1.66)	0.385	0.770	1.02 (0.50, 2.07)	1.09 (0.84, 1.42)	1.08 (0.76, 1.53)	1.17 (0.82, 1.67)	1.16 (0.82, 1.63)
Copper	1.01 (0.93, 1.10)	0.835	0.927	–	–	–	1.04 (0.83, 1.30)	–
Folate	0.94 (0.60, 1.47)	0.778	0.927	–	–	–	1.15 (0.90, 1.48)	–
Iron	1.16 (1.05, 1.29)	0.006	0.028	1.28 (1.00, 1.63)	1.18 (1.03, 1.35)	1.27 (1.07, 1.51)	1.20 (1.06, 1.35)	1.24 (1.11, 1.39)
Magnesium	1.09 (0.18, 6.71)	0.927	0.927	1.17 (0.60, 2.31)	1.07 (0.87, 1.32)	1.06 (0.83, 1.37)	1.20 (1.06, 1.35)	1.22 (0.19, 7.77)
Phosphorus	0.90 (0.55, 1.47)	0.676	0.927	0.98 (0.56, 1.69)	0.96 (0.54, 1.69)	0.96 (0.54, 1.69)	0.90 (0.55, 1.48)	0.96 (0.79, 1.17)
Selenium	1.11 (1.03, 1.20)	0.005	0.028	0.81 (0.52,1.25)	1.12 (1.03, 1.21)	1.18 (1.05, 1.32)	1.12 (1.03, 1.21)	1.11 (1.04, 1.20)
Vitamin B6	1.00 (0.98, 1.02)	0.868	0.927	–	–	–	–	–
Vitamin B12	1.07(1.03, 1.13)	0.002	0.028	1.10 (0.94, 1.28)	1.07 (0.99, 1.14)	1.07 (0.94, 1.21)	1.08 (1.02, 1.13)	1.07 (1.03, 1.12)
Vitamin C	0.96 (0.82, 1.14)	0.661	0.927	0.93 (0.70, 1.23)	0.94 (0.76, 1.15)	1.05 (0.82, 1.34)	0.97 (0.82, 1.16)	1.00 (0.87, 1.14)
Vitamin D	0.96 (0.83, 1.12)	0.635	0.927	1.03 (0.82, 1.29)	0.98 (0.83, 1.15)	0.99 (0.86, 1.15)	0.96 (0.82, 1.13)	0.96 (0.90, 1.02)
Vitamin K1	0.93 (0.85, 1.02)	0.126	0.353	0.84 (0.65, 1.09)	0.94 (0.84, 1.05)	0.97 (0.86, 1.10)	0.93 (0.84, 1.02)	–
Zinc	0.95 (0.87, 1.03)	0.185	0.432	–	–	–	0.95 (0.87, 1.02)	–

^a^P value for inverse-variance weighted.

### Significant associations of circulating iron, selenium and vitamin B12 with risk of NAFLD

Genetically predicted circulating iron, selenium and vitamin B12 were significantly associated with the increased risk of NAFLD in the combined dataset (Fig. 2). The scatter plots for the effect of IVs on the three micronutrients and NAFLD are depicted in Supplementary Fig. 1. For one SD increase in genetically predicted iron and selenium levels, the combined odds ratio (OR) of NAFLD were 1.16 (95% confidence interval (CI) 1.05–1.29; *P* = 0.006,* P*_FDR_ = 0.028), 1.11 [95% CI 1.03–1.20; *P* = 0.005,* P*_FDR_ = 0.028] and 1.08 (95% CI 1.03–1.13; *P* = 0.002, *P*_FDR_ = 0.028), respectively (Table 1). Such results were consistent in the weighted median (OR 1.18, 95% CI 1.03–1.35; *P* = 0.016 for iron; OR 1.12, 95% CI 1.03–1.21; *P* = 0.007 for selenium, OR 1.08, 95% CI 1.03–1.14; *P* = 0.003 for vitamin B12), simple median (OR 1.27, 95% CI 1.07–1.51; *P* = 0.006 for iron; OR 1.18, 95% CI 1.05–1.32; *P* = 0.005 for selenium) and maximum-likelihood method (OR 1.20, 95% CI 1.06–1.35; *P* = 0.003 for iron; OR 1.12, 95% CI 1.03–1.21; *P* = 0.009 for selenium). Furthermore, MR-Egger regression did not suggest evidence of potential directional pleiotropy (iron, *P* = 0.960; selenium, *P* = 0.722; vitamin B12, *P* = 0.503) (Table S4). No heterogeneity was found in circulating iron, selenium and vitamin B12 using Cochran’s Q statistic (*P* > 0.05) (Table S4).

### Nominal associations of circulating β‑carotene with risk of NAFLD

Genetically predicted circulating β‑carotene levels were inversely associated with NAFLD risk in replication 2 using the IVW method, and the associations were directionally consistent in the other two databases (Fig. 2). For one SD increase in genetically predicted circulating β‑carotene levels, the combined OR of NAFLD was 0.81 (95%CI 0.66–0.99; *P* = 0.047,* P*_FDR_ = 0.164) (Table 1). The associations were concordant in the weighted median (OR 0.82, 95% CI 0.68–0.98; *P* = 0.033) and maximum-likelihood method (OR 0.81, 95% CI 0.67–0.99, 1.28; *P* = 0.034) (Table 1). There was no indication for potential directional pleiotropy effects as assessed by the intercept of the MR-Egger regression (*P* = 0.123) (Table S4). Cochran’s Q statistic did not suggest heterogeneity of effect estimates (*P* > 0.05) (Table S4).

### Nonsignificant associations

We observed little evidence that the circulating concentrations of the other micronutrients were associated with the risk of NAFLD. Genetically predicted vitamin D level was associated with reduced NAFLD risk in replication stage 1 (OR 0.77, 95% CI 0.60–1.00; *P* = 0.047), but these associations were not significant in the meta-analysis (Fig. 2).

## Discussion

In this MR analysis, we provided evidence that genetically predicted high iron, selenium and vitamin B12 levels were associated with elevated NAFLD risk, and circulating β‑carotene levels were suggestively associated with reduced risk of NAFLD. However, little evidence was observed for the association of other micronutrients with the risk of NAFLD.

### Circulating iron levels were associated with increased risks of NAFLD

Accumulating studies have investigated the association between iron status and risk of NAFLD^41,42^. Our study supports the findings of previous studies that circulating iron status increase the risk of developing NAFLD using larger summary statistics. In terms of the mechanisms of the association between iron and NAFLD, the pathogenesis is related to altered regulation of iron transport associated with steatosis, insulin resistance, oxidative stress and subclinical inflammation^43^. Also, dysmetabolic iron overload syndrome may facilitate the evolution to type 2 diabetes by altering beta-cell function, the progression of cardiovascular disease by contributing to the recruitment and activation of macrophages within arterial lesions, and the natural history of liver disease by inducing oxidative stress in hepatocytes, activation of hepatic stellate cells, and malignant transformation by promotion of cell growth and DNA damage^43^. Interestingly, a recent study suggested there was a significant cross-talk among gut microbiota, iron status, and liver fat accumulation. The mechanism was speculated to be microbiome- and iron-linked metabolomic and transcriptomic signatures involving imbalances in gluconeogenic metabolites, ketone bodies, and cellular transport, which altogether modulate liver fat accumulation^44^. Besides, establishing the exact function of abnormal iron status in the regulation of NAFLD is crucial from a clinical perspective. Previous evidence has indicated that NAFLD subjects might suffer from iron overload^45,46^. Moreover, studies have already shown that higher iron status is associated with an increased risk of NAFLD^47,48^. Accurate assessment of iron levels and iron-related markers can serve as diagnostic tools, allowing for the early identification of at-risk individuals and the controling of disease progression. Low-iron diet therapy has been proven to be effective in reducing hepatitis progression ^49^, thus iron restriction could be useful as a novel therapeutic approach for NAFLD.

### Circulating selenium levels were associated with increased risks of NAFLD

For selenium, a positive association of high circulating selenium levels with risk of NAFLD has been found in some^50–52^ but not all observational study^53^. Leveraging genetic variants as instrument variables for selenium, our study found that genetically predicted higher circulating selenium levels were robustly associated with increased risk of NAFLD. Increased selenium levels can elevate the selenomethionine, which might be metabolized to selenols, selenohomocysteine and selenocysteine via the methionine cycle and the trans-sulfuration pathway. These metabolites of selenomethionine can induce oxidative stress via generating superoxide radicals, which plays a role in the development and progression of NAFLD^50,54^. In addition, evidence from studies in rodent models demonstrated that selenium exposure was more potent in inducing liver damage by activating inflammation and the liver with infiltration by inflammatory cells, increasing hepatic enzymes and accumulation of glycogen and lipid^55,56^. Taken together, mechanisms behind the harmful effect of high iron and selenium status on risk of NAFLD remain to be elucidated and need further investigation, and more sophisticated clinical trials should be carried out to corroborate our findings and give proper diet guidance. Elucidating the impact of selenium on NAFLD will be of significant values in clinical applications. Previous cross-sectional study has confirmed that the mean selenium intake in NAFLD patients was higher than the recommended dietary allowance (RDA) level^57^. According to most of the evidence, excessive intake of selenium is associated with the risk of NAFLD. From a clinical standpoint, assessing the selenium status of NAFLD patients and correcting selenium homeostasis can potentially prevent NAFLD. A recent meta-analysis revealed a positive correlation between selenium exposure and diabetes in both epidemiological and experimental studies ^58^. Increased selenium intake elevates expression of intracellular selenoprotein levels, resulting in heightened reactive oxygen species (ROS) production^59,60^, which was also served as a potential pathogenic mechanism for NAFLD ^61,62^.

### Circulating vitamin B12 levels were associated with increased risks of NAFLD

Previous observational studies on the relationship between vitamin B12 concentrations and NAFLD were controversial. Recently, a meta-analysis synthesizeing eight cross-sectional and case–control studies found that there was no significant difference in vitamin B12 concentrations between NAFLD cases and individuals without NAFLD^63^. A subsequent study using National Health and Nutrition Examination Survey (NHANES) 1999–2004 data also failed to find an association between vitamin B12 and risk of NAFLD^64^. However, another NHANES 2017–2018 study found linear positive correlations between vitamin B12 and hepatic steatosis and fibrosis^8^. Interestingly, our study utilized large-scaled GWAS encompassing over 12,000 NAFLD patients and 820,000 controls, and demonstrated a significant impact of genetic predicted higher vitamin B12 levels on increased NAFLD risk, overcoming bias to a greater extent such as small sample sizes and confounders in observational studies. Higher serum levels of vitamin B12 were positively correlated with the severity of steatosis and fibrosis in 614 Brazilian patients^65^. Another study conducted among 120 Jordanians also suggested that NAFLD patients consumed higher dietary intakes of vitamin B12 than controls^66^. Since the small number of participants in previous studies, more research is needed on the mechanisms underlying vitamin B12 and the increased risk of NAFLD in the future. Though previous research did not clearly establish a relationship between vitamin B12 and NAFLD, the present study provides newfound insight into the role of vitamin B12 in NAFLD etiology.

### Circulating β‑carotene levels were associated with decreased risks of NAFLD

A recent MR study on dietary sources of antioxidants and NAFLD failed to find an inverse association between β‑carotene and NAFLD^67^, possibly due to low statistical power to detect weak associations. Interestingly, we detected that genetically predicted circulating beta-carotene levels are likely to be negatively associated with NAFLD risk by using a larger summary statistic of NAFLD, which is consistent with other epidemiological studies that demonstrate a negative association between serum β‑carotenoid levels and NAFLD^68–70^. Higher β‑carotenoid intake may reduce risk for NAFLD and the progression of simple hepatic steatosis to nonalcoholic steatohepatitis, through several different pathways, including the attenuation of inflammation and oxidative stress in the liver, with downstream effects on secretion of pro-inflammatory cytokines by hepatic macrophages, immune infiltration, and insulin sensitivity^71^. Furthermore, β-carotene has also been reported to have preventive effects on hepatic inflammation, fibrosis and cirrhosis in animal models^72^. Regarding to the association did not reach significance after FDR correction, thus, we still emphasize that independent MR studies and large prospective trials are needed to validate our findings. Furthermore, determining the precise role of β-carotene in the regulation of NAFLD is of great benefits from a clinical perspective. We have noted that more than half of the NAFLD patients presented low β-carotene consumption in a cross-sectional study^73^. Moreover, dietary β-carotene can prevent NAFLD by down regulating inflammation^74^. To be noted, fucoxanthin, a carotenoid mainly found in brown seaweed, has recently entered clinical trials as a potential treatment for NAFLD^75^. Another randomized controlled clinical trial suggested that the administration of fucoxanthin combined with fucoidan for 6 months attenuated hepatic lipotoxicity in 21 patients with NAFLD ^76^. The present evidence can inform dietary advice and supplemental interventions for individuals at risk of NAFLD, offering a non-invasive and cost-effective means of prevention.

### Insignificant associations between other micronutrients and NAFLD

For other micronutrients, three recent MR studies on the association of vitamin D and NAFLD yielded inconsistent. In our study, though vitamin D concentrations were observed to be associated with a reduced risk of NAFLD in the replication dataset with a smaller sample size, this association was not observed in the other datasets. The relationship between vitamin D and pathogenesis and progression of NAFLD remains unclarified and may be complex. It is noteworthy that the observation that genetic variations of vitamin D-related genes were associated with presence, severity and response to treatment of NAFLD provides additional insights, as it may indicate interindividual differences in the responsiveness to vitamin D on NAFLD^77^. Therefore, we considered that the use of vitamin D was supposed to be selective, and further population categories should be performed.

In the current MR study, we observed little evidence of associations between other genetically predicted circulating micronutrients concentrations with risk of NAFLD. However, we cannot exclude the presence of a potential causal association. For example, the B vitamins, such as folate and vitamins B6, play vital roles in the metabolism of homocysteine^78^. Deficiency of either of these B vitamins can lead to an elevated circulating level of total homocysteine (tHcy), which has been implicated in the development of NAFLD^79,80^. Further studies found that folate could have potential for the prevention or treatment of NAFLD^81^. However, these associations were not observed in our study, thus, larger GWASs are needed to better understand the regulation of micronutrients and to better define instrumental variables for MR analysis.

### Strengths and limitations of this study

The main strength in this study is the MR design, which can reduce confounding and reverse causality to a large extent. We explored associations in three independent separate to examine the consistency, and then combined the associations from three data sources to increase the number of cases. Together with larger sample compared to previous MR studies, our established associations should be better powered even though we might overlook weak associations. The results remained overall consistent across several sensitivity analyses. Limitations need consideration when interpreting our results. First, the major issue for MR study is horizontal pleiotropy that means selected genetic IVs influence the risk of outcome not via the exposure but other alternative pathways. Although we could not completely rule out the possibility that our findings might be biased by horizontal pleiotropy, our results remained consistent across several sensitivity analyses and the MR-Egger detected limited evidence in support of strong pleiotropic effects. Second, associations for micronutrients differed between the discovery and replication datasets, which might be caused by differences in NAFLD definition might cause heterogeneity in meta-analysis of associations across used data sources. In addition, MR relies on the assumption of a linear relationship between the exposure and the outcome. This assumption may not hold true in all cases, especially if there are non-linear associations involved. Micronutrients might have optimal ranges for beneficial effects, and deviations from these ranges could lead to different health outcomes. Failing to account for non-linearity in this relationship may result in biased causal estimates. Furthermore, MR considers the lifetime effect of micronutrients status by using genetic variants as IVs^82^, thus our MR results should not be extrapolated to extremes of micronutrients status.

## Conclusions

In conclusion, this MR study observed that genetically predicted β‑carotene levels were inversely associated with NAFLD risk, whereas the genetically predicted iron, selenium and vitamin B12 levels were positively associated with NAFLD risk. These findings are promising given the limited therapeutic options for NAFLD, in that identification of modifiable lifestyle factors provides an opportunity to limit or prevent the disease and its progression.

### Supplementary Information

Supplementary Figure S1.

Supplementary Tables.

Supplementary Legends.

## Data Availability

All the data used in the present study had been publicly available, and the source of the data had been described in the main text. All analyzed data are available from the corresponding author upon reasonable request.

## Abbreviations

NAFLD: Nonalcoholic fatty liver disease
GWAS: Genome-wide association study
OR: Odd ratio
Cl: Confidence interval
FDR: False discovery rate
MR: Mendelian randomization
IV: Instrumental variable
BMI: Body mass index
SNP: Single nucleotide polymorphism
IVW: Inverse-variance weighted
NHANES: National Health and Nutrition Examination Survey
tHcy: Total homocysteine
