# Supplementary figures and images for: Genetically determined circulating micronutrients and the risk of nonalcoholic fatty liver disease
Source: Sci Rep. 2024 Jan 11;14:1105. doi: 10.1038/s41598-024-51609-3 (PMC10784479; doi:10.1038/s41598-024-51609-3)

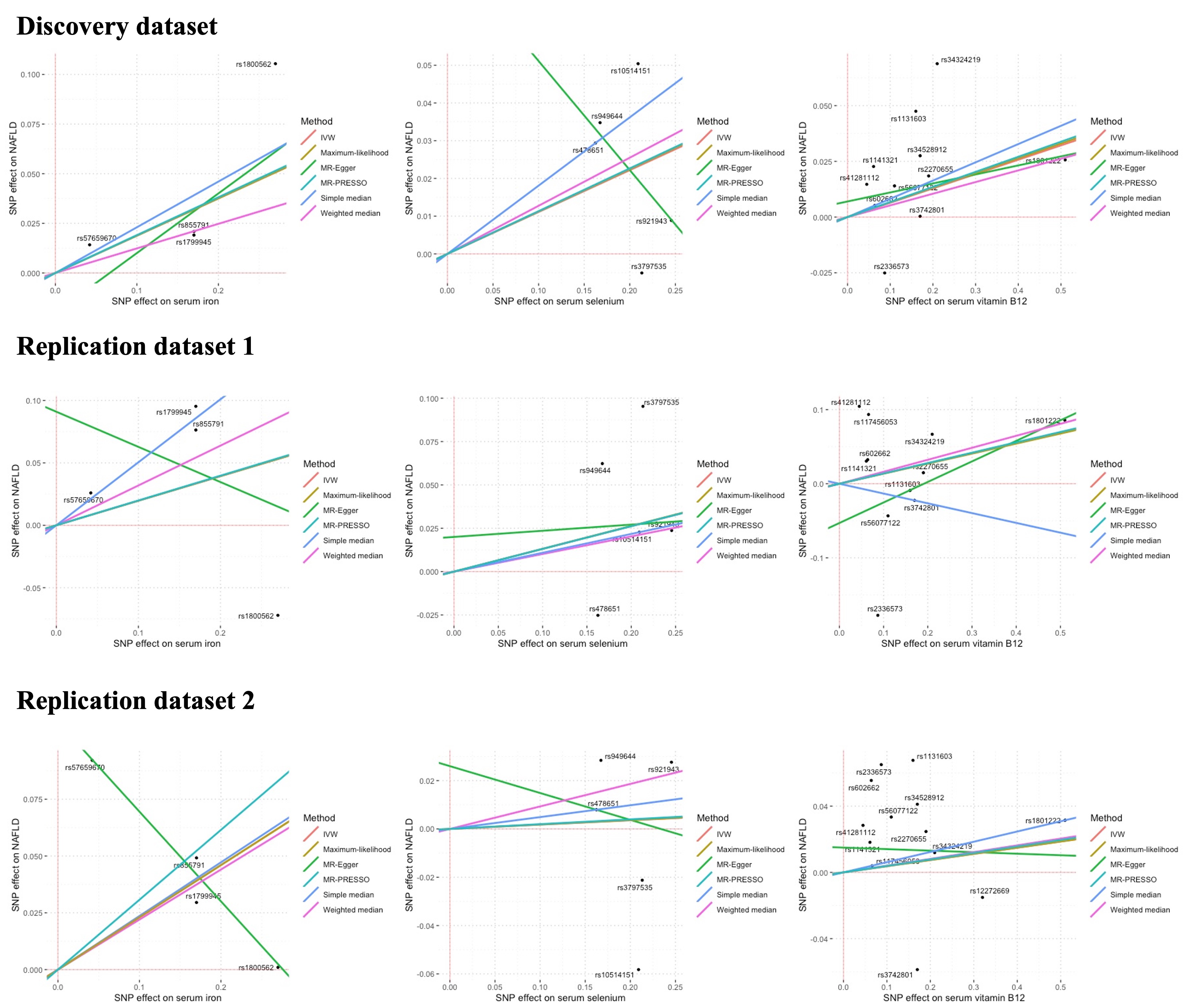

Supplement: Supplementary file 1 — Supplementary Figure S1. [file 41598_2024_51609_MOESM1_ESM.jpg]
