# Supplementary material for: Genetically determined circulating micronutrients and the risk of nonalcoholic fatty liver disease
Source: Sci Rep. 2024 Jan 11;14:1105. doi: 10.1038/s41598-024-51609-3 (PMC10784479; doi:10.1038/s41598-024-51609-3)
Supplement: Supplementary file 3 — Supplementary Legends. [file 41598_2024_51609_MOESM3_ESM.docx]

**Supplementary Figure 1.** Scatter plots of the casual association of genetically predicted iron, selenium and vitamin B12 with NAFLD for each dataset.
